# The *Arabidopsis thaliana* chloroplast division protein FtsZ1 counterbalances FtsZ2 filament stability *in vitro*

**DOI:** 10.1016/j.jbc.2021.100627

**Published:** 2021-04-02

**Authors:** Katie J. Porter, Lingyan Cao, Yaodong Chen, Allan D. TerBush, Cheng Chen, Harold P. Erickson, Katherine W. Osteryoung

**Affiliations:** 1 Department of Plant Biology, Michigan State University, East Lansing, Michigan, USA; 2 Department of Cell Biology, Duke University School of Medicine, Durham, North Carolina, USA

**Keywords:** *Arabidopsis thaliana*, chloroplast, cytoskeleton, GTPase, plant biochemistry, FtsZ dynamics, At, *Arabidopsis thaliana*, Cc, critical concentration, CTP, conserved C-terminal peptide, FRAP, fluorescent recovery after photobleaching, FtsZ, filamenting temperature-sensitive Z, Gs, *Galdieria sulphuraria*, IPTG, isopropyl-β-D-1-thiogalactopyranoside, LS, light scattering, SD, standard deviation, TEM, transmission electron microscopy, TP, chloroplast transit peptide, Mt, *Medicago truncatula*, LSB, low salt buffer

## Abstract

Bacterial cell and chloroplast division are driven by a contractile “Z ring” composed of the tubulin-like cytoskeletal GTPase FtsZ. Unlike bacterial Z rings, which consist of a single FtsZ, the chloroplast Z ring in plants is composed of two FtsZ proteins, FtsZ1 and FtsZ2. Both are required for chloroplast division *in vivo*, but their biochemical relationship is poorly understood. We used GTPase assays, light scattering, transmission electron microscopy, and sedimentation assays to investigate the assembly behavior of purified *Arabidopsis thaliana* (At) FtsZ1 and AtFtsZ2 both individually and together. Both proteins exhibited GTPase activity. AtFtsZ2 assembled relatively quickly, forming protofilament bundles that were exceptionally stable, as indicated by their sustained assembly and slow disassembly. AtFtsZ1 did not form detectable protofilaments on its own. When mixed with AtFtsZ2, AtFtsZ1 reduced the extent and rate of AtFtsZ2 assembly, consistent with its previously demonstrated ability to promote protofilament subunit turnover in living cells. Mixing the two FtsZ proteins did not increase the overall GTPase activity, indicating that the effect of AtFtsZ1 on AtFtsZ2 assembly was not due to a stimulation of GTPase activity. However, the GTPase activity of AtFtsZ1 was required to reduce AtFtsZ2 assembly. Truncated forms of AtFtsZ1 and AtFtsZ2 consisting of only their conserved core regions largely recapitulated the behaviors of the full-length proteins. Our *in vitro* findings provide evidence that FtsZ1 counterbalances the stability of FtsZ2 filaments in the regulation of chloroplast Z-ring dynamics and suggest that restraining FtsZ2 self-assembly is a critical function of FtsZ1 in chloroplasts.

Chloroplasts, the photosynthetic organelles in plants, arose from the endosymbiosis of a free-living cyanobacterium (1). Like bacteria, chloroplasts divide by binary fission, ensuring they are faithfully inherited during cytokinesis (2, 3, 4). While the chloroplast and bacterial division complexes are quite different, a key component they share in common is the tubulin-like protein filamenting temperature-sensitive Z (FtsZ). FtsZ is a self-assembling cytoskeletal protein that assembles into a membrane-tethered “Z ring” at the nascent division site inside the cell or organelle (5, 6, 7, 8, 9, 10, 11, 12, 13, 14, 15). Z-ring formation initiates assembly of the entire bacterial or chloroplast division complex, and the subsequent constriction of the Z ring helps drive membrane invagination during division (16). Recent models suggest that bacterial Z rings are composed of single-stranded polymers, called protofilaments, which overlap and may interact laterally to encircle the division site (10, 11). Bacterial protofilaments are dynamic; they continuously exchange subunits with a soluble pool of FtsZ monomers and treadmill at steady state, meaning that subunits associate onto one end of the protofilament and dissociate from the other end (7, 11, 12, 13, 17, 18, 19, 20, 21, 22, 23). Subunit exchange (turnover) is critical for Z-ring remodeling and cell division *in vivo*. Chloroplast Z rings also exhibit dynamic subunit exchange (4, 24, 25, 26, 27), though their substructure is unknown. One study suggests that chloroplast FtsZs may also treadmill (27), but this has not been explored.

Extensive *in vitro* investigation has revealed that the dynamics of bacterial FtsZ protofilaments is an emergent property of their GTPase activity. Such studies have demonstrated that FtsZ polymerization is GTP-dependent because GTP-bound monomers assemble onto a growing protofilament. However, the GTPase active site is formed in the longitudinal interface between two subunits. Therefore, GTP hydrolysis requires oligomerization (28, 29). Hydrolysis weakens the interface and facilitates dissociation of GDP-bound subunits from protofilament ends. Following nucleotide exchange, subunits recycle back onto protofilaments (30). Recent *in vitro* and modeling studies have explained how GTP hydrolysis, coupled with a conformational change in the FtsZ subunit, can lead to preferential loss of subunits from one end of the protofilament and addition onto the other end, producing treadmilling (19, 23). Thus cycles of assembly and GTPase-dependent subunit dissociation drive emergent protofilament dynamics independently of any other proteins (11, 13). However, because FtsZ is self-assembling, the formation of Z rings in both bacteria and chloroplasts is confined to the division site *in vivo* primarily by negative regulatory systems that hinder FtsZ assembly elsewhere (4, 31, 32, 33). *In vitro* study of FtsZ proteins has therefore not only revealed their intrinsic self-assembly and dynamic properties, but has also been crucial for understanding the functions of the many factors that regulate Z-ring assembly and dynamics *in vivo*.

Unlike bacterial Z rings, which are composed of a single FtsZ, chloroplast Z rings in plants are more complex because they consist of two distinct types of FtsZ called FtsZ1 and FtsZ2. FtsZ1 and FtsZ2 presumably arose through ancient duplication of a single *FtsZ* gene acquired from the cyanobacterial endosymbiont and have been conserved throughout green algae and land plants (2, 34, 35). Both proteins are now encoded in the nucleus, targeted across the two chloroplast envelope membranes by N-terminal targeting sequences called transit peptides, and released as soluble proteins into the stroma, the topological equivalent of the bacterial cytoplasm (15, 36, 37). Knockout of either *FtsZ1* or *FtsZ2* impairs chloroplast division, resulting in reduced numbers of enlarged chloroplasts in leaf cells, and genetic analysis in the model plant *Arabidopsis thaliana* has established that both proteins are required for normal Z-ring function and chloroplast division (35, 38, 39, 40). Several lines of evidence imply that FtsZ1 and FtsZ2 interact directly and most likely coassemble. In *A. thaliana* (At), endogenous AtFtsZ1 and AtFtsZ2 consistently colocalize, not only to Z rings in wild-type plants, but also to abnormal FtsZ structures observed in various chloroplast division mutants (6, 15). Additionally, fluorescently tagged forms of AtFtsZ1 and AtFtsZ2 tightly colocalize in heterologous systems (24, 25) and direct evidence of coassembly was shown using chimeric AtFtsZ1/AtFtsZ2 proteins (27). Colocalization of FtsZ1 and FtsZ2 in other species also supports their coassembly (6).

FtsZ1 and FtsZ2 differ in several important ways. Both possess a highly conserved globular core region responsible for GTP binding and hydrolysis in all FtsZs, flanked by more variable N- and C-terminal regions (41, 42, 43, 44, 45). However, only FtsZ2 retains a conserved peptide near the C-terminus (conserved C-terminal peptide, CTP) that in bacteria mediates Z-ring tethering to the membrane through interaction with membrane proteins (2, 7, 9, 13, 44, 45, 46, 47, 48) (Fig. 1*A*). Similarly, the FtsZ2 CTP tethers the chloroplast Z ring to the inner envelope membrane through interaction with plant-specific membrane proteins (40, 49, 50). FtsZ1 lacks the CTP and does not interact directly with any known membrane protein. Therefore, its localization to the Z ring is presumed to be a consequence of its coassembly with FtsZ2 (49, 50). The two proteins also differ in their dynamic properties, as shown by fluorescence recovery after photobleaching (FRAP) experiments in which the AtFtsZ proteins were expressed in heterologous yeast systems. While both proteins form homopolymeric filaments and/or rings that undergo subunit exchange in such systems, AtFtsZ2 filaments are much less dynamic than AtFtsZ1 or coassembled filaments (24, 27). These studies, in combination with mutant analysis in *Arabidopsis*, have led to proposals that FtsZ2 imparts structural stability to the Z ring while FtsZ1 opposes this stability and promotes Z-ring turnover dynamics (24, 26, 27, 39). Complementary *in vitro* studies are essential for further understanding of how these proteins cooperate biochemically in the chloroplast Z ring.

**Figure 1:**
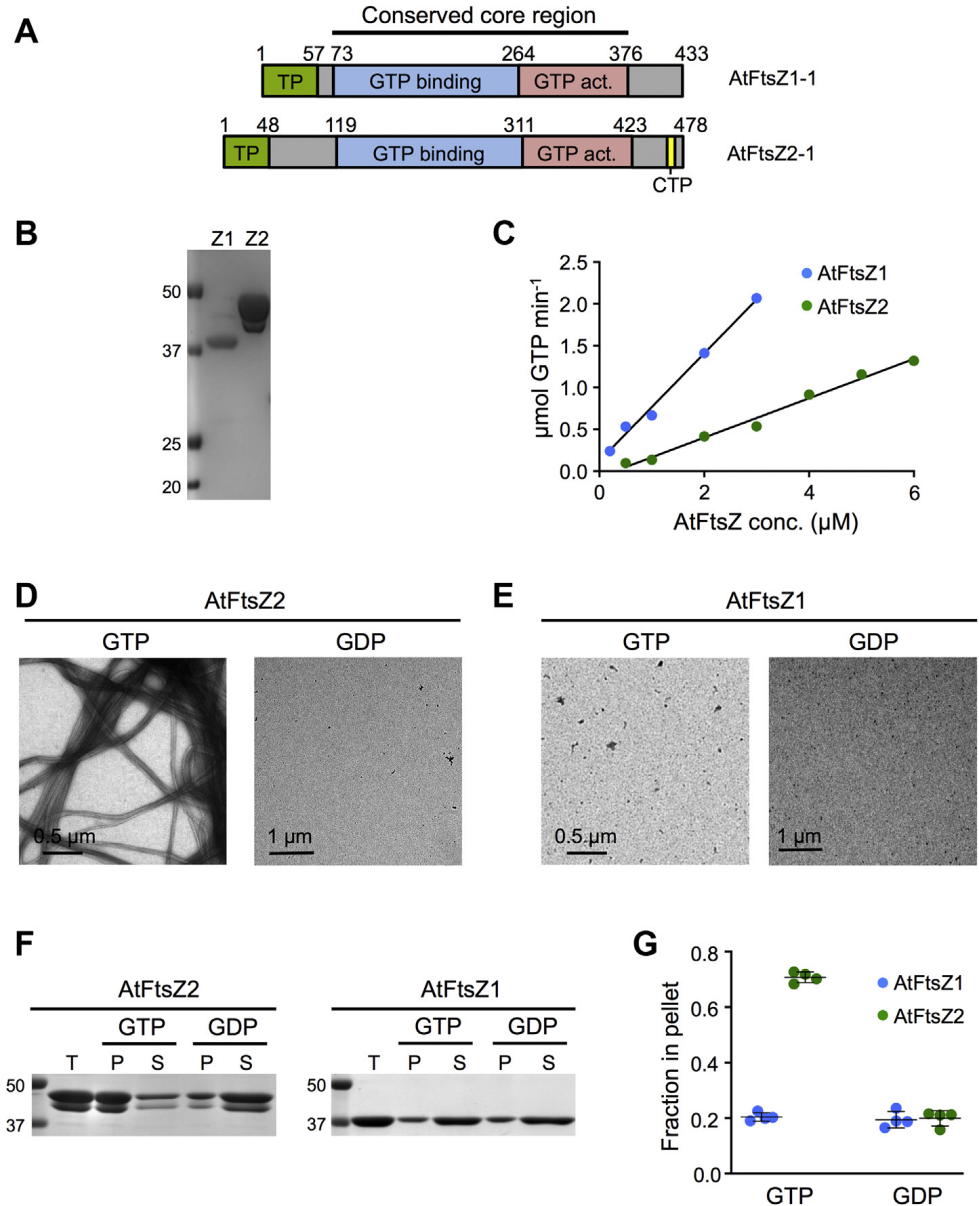
**AtFtsZ1 and AtFtsZ2 hydrolyze GTP, but only AtFtsZ2 exhibits GTP-dependent assembly *in vitro*.***A*, schematics of the complete *AtFtsZ1-1* (At5g55280) and *AtFtsZ2-1* (At2g36250) gene products. The constructs used in this study encoded the full-length mature proteins lacking their transit peptides (AtFtsZ1, aa 58–433; AtFtsZ2, aa 49–478) or the conserved core regions (AtFtsZ1_core_, aa 73–376; AtFtsZ2_core_, aa 119–423). The latter are composed of only the GTP binding (*blue*) and GTPase activating (*pink*) domains. TP, chloroplast transit peptide (*green*); CTP, AtFtsZ2 C-terminal peptide (aa 459–467, *yellow*). *B*, SDS-PAGE of purified AtFtsZ1 (Z1) and AtFtsZ2 (Z2) proteins. The gel was stained with Coomassie. Note that purified AtFtsZ2 runs as a doublet, as does endogenous AtFtsZ2-1 in plant extracts (60). Markers (kDa) are shown in the *left*. *C*, GTPase activities assayed in 500 μM GTP at 25 °C at protein concentrations ranging from 0.2 to 3 μM for AtFtsZ1 (*blue*) and from 0.5 to 6 μM for AtFtsZ2 (*green*). A representative set of assays is shown. The GTPase activity is the slope of the regression line above the Cc (Table 1). *D* and *E*, negative-stain TEM of 3 μM AtFtsZ2 or AtFtsZ1 incubated for 5 min at room temperature after addition of 500 μM GTP or GDP. Scale bars are as indicated. *D*, AtFtsZ2. The average width of AtFtsZ2 bundles in GTP was 60.15 ± 20.5 nm (SD; n = 32); Fig. S5. *E*, AtFtsZ1. *F* and *G*, sedimentation assays. Reactions containing 5 μM AtFtsZ2 or AtFtsZ1 were incubated for 30 min at room temperature after addition of 500 μM GTP or GDP, then centrifuged at 80,000*g* for 30 min at 25 °C. *F*, SDS-PAGE of proteins in the total (T), pellet (P), and supernatant (S) fractions. Representative gels stained with Coomassie are shown. Markers (kDa) are shown on the *left*. *G*, fraction of AtFtsZ protein in the pellet (n = 4).

Here, we used purified AtFtsZ1 and AtFtsZ2 to test their self-assembly behavior *in vitro* and elucidate how their interactions contribute to their cellular roles. Toward this end we compared the GTPase activities, formation of protofilaments, and assembly kinetics of AtFtsZ1 and AtFtsZ2 separately and in mixture. We provide biochemical evidence that FtsZ1 counterbalances the stabilizing properties of FtsZ2 by restraining its assembly into protofilaments.

## Results

### AtFtsZ1 and AtFtsZ2 exhibit distinct assembly properties

In a previous analysis, bacterially expressed AtFtsZ proteins were insoluble and had to be renatured (51). Here we optimized expression and purification of soluble, His-tagged AtFtsZ1 and AtFtsZ2 (Fig. 1*B*) to investigate their *in vitro* enzymatic and assembly properties. Proteins were expressed without their transit peptides (Fig. 1*A*) because *in vivo* these sequences are cleaved upon organelle import (15, 36, 37). Although GTP-dependent assembly of a similar AtFtsZ2 construct has been reported (52), to date no *in vitro* study has characterized the equivalent soluble AtFtsZ1 construct alone or in combination with AtFtsZ2, which is important for fully understanding how they cooperate in Z-ring dynamics.

**Figure 2:**
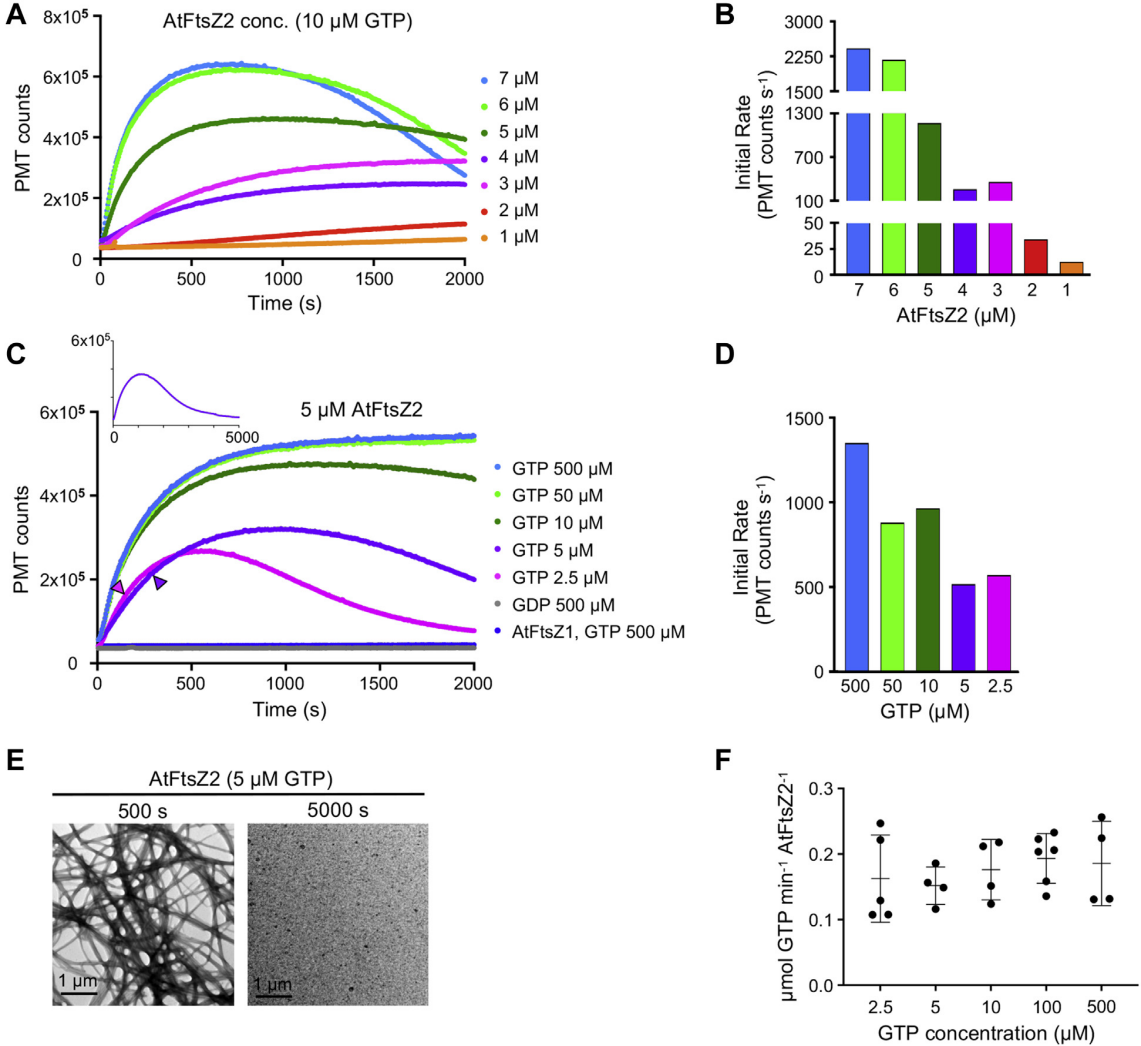
**AtFtsZ2 assembles stable protofilaments.** Assembly reactions were performed at room temperature, initiated by addition of nucleotide, and repeated at least twice with similar results. *A*, assembly of AtFtsZ2 monitored by light scattering (LS) at the indicated protein concentrations after addition of 10 μM GTP. *B*, initial rates of AtFtsZ2 assembly for the LS traces in *A* (see Experimental procedures; initial rates determined for other LS replicates showed similar trends). *C*, LS assays of 5 μM AtFtsZ2 after addition of GTP or GDP at the indicated nucleotide concentrations. *Arrowheads* show the predicted times of GTP depletion for the reactions initiated with 5 μM GTP (*purple*) and 2.5 μM GTP (*pink*). *Inset* displays an extended assay initiated with 5 μM GTP for 5000 s. The y-axis (PMT counts) is the same as in the larger plot, while each tick on the x-axis denotes 1000 s. LS of 5 μM AtFtsZ1 after addition of 500 μM GTP is also shown (*dark blue*). *D*, initial rates of 5 μM AtFtsZ2 assembly for the LS traces in *C*. *E*, negative-stain TEM of 5 μM AtFtsZ2 incubated for 500 s (*left*) or 5000 s (*right*) after addition of 5 μM GTP. *F*, GTPase activity of AtFtsZ2 at different GTP concentrations. For assays in 2.5, 5, 10, 100, and 500 μM GTP, n = 5, 4, 4, 6, and 4, respectively. There were no significant differences between any two means (*p* > 0.7) as determined by a one-way ANOVA using Tukey’s multiple comparison test.

The GTPase activity of AtFtsZ2 at 25 °C was 0.22 GTP min^−1^ FtsZ^−1^ (slope of the line in Fig. 1*C*; Table 1), slightly lower than previously reported (52). The critical concentration (Cc) for AtFtsZ2 assembly based on GTPase activity (53, 54) was 0.37 μM, meaning that below 0.37 μM AtFtsZ2, the GTPase activity was ∼0, and above 0.37 μM GTPase increased linearly with concentration (Fig. 1*C*, Table 1). Transmission electron microscopy (TEM) showed that AtFtsZ2 assembles into protofilament bundles with GTP but not GDP (Fig. 1*D*). Sedimentation assays confirmed that AtFtsZ2 assembly is GTP-dependent (52), as indicated by the substantial increase in the proportion of AtFtsZ2 in the pellet fraction in GTP *versus* GDP (Fig. 1, *F* and *G*). Consistent with these results 90° light scattering (LS) assays demonstrated an increase in the LS signal only in GTP (Fig. 2*C*).

**Table 1:** GTPase activities and critical concentrations for AtFtsZ1, AtFtsZ2, AtFtsZ1_D275A_, AtFtsZ1_core_, and AtFtsZ2_core_, and their mixtures at various ratios

AtFtsZ components	GTPase activity (GTP min^−1^ FtsZ^−1^)	Cc (μM)
AtFtsZ1^a^^,^^b^^,^^c^	0.52 ± 0.16 (n = 10)	−0.031 ± 0.39
AtFtsZ2^a^^,^^d^	0.22 ± 0.03 (n = 6)	0.37 ± 0.20
AtFtsZ2:AtFtsZ1 (1:0.1)	0.29 ± 0.04 (n = 4)	0.43 ± 0.59
AtFtsZ2:AtFtsZ1 (1:0.2)	0.26 ± 0.05 (n = 4)	0.19 ± 0.31
AtFtsZ2:AtFtsZ1 (1:0.5)	0.27 ± 0.03 (n = 4)	0.043 ± 0.26
AtFtsZ2:AtFtsZ1 (1:1)	0.36 ± 0.10 (n = 4)	−0.075 ± 0.30
AtFtsZ1_D275A_^c^	0.079 ± 0.008 (n = 3)	0.21 ± 0.32
AtFtsZ1_core_^e^^,^^b^	0.27 ± 0.03 (n = 7)	0.39 ± 0.24
AtFtsZ2_core_^e^^,^^d^	0.20 ± 0.03 (n = 6)	0.57 ± 0.33
AtFtsZ2_core_:AtFtsZ1_core_ (1:0.1)	0.15 ± 0.009 (n = 3)	0.18 ± 0.40
AtFtsZ2_core_:AtFtsZ1_core_ (1:0.2)	0.15 ± 0.02 (n = 3)	0.20 ± 0.22
AtFtsZ2_core_:AtFtsZ1_core_ (1:0.5)	0.16 ± 0.002 (n = 3)	0.24 ± 0.12
AtFtsZ2_core_:AtFtsZ1_core_ (1:1)	0.19 ± 0.009 (n = 3)	0.13 ± 0.15

GTPase activities were calculated based on the total AtFtsZ concentration. Values represent the average of the indicated number of assays (n) ± SD. All reactions were performed with 500 μM GTP at 25 °C using a regenerative system that maintained the GTP at this concentration (64). *p* values for statistical comparisons between GTPase activities and critical concentrations (Cc) of individual proteins are shown in the footnotes.

a *p* = 0.0005 for GTPase; *p* = 0.0362 for Cc.

b *p* = 0.0009 for GTPase; *p* = 0.0214 for Cc.

c *p* = 0.0007 for GTPase; no significant difference for Cc.

d No significant difference for GTPase or Cc.

e *p* = 0.0017 for GTPase; no significant difference for Cc.

LS has been used extensively to understand the kinetics of the assembly and disassembly of bacterial FtsZs, which both contribute to overall protofilament dynamics (55, 56). The LS signal reflects the mass, size, and shape of the ensemble of assembled structures and is influenced by protofilament bundling. In general, a signal increase indicates net assembly, a plateau indicates a steady state where overall assembly remains constant though subunit exchange continues, and a decrease indicates net disassembly, providing a qualitative measure of assembly (54, 55, 56, 57, 58, 59). We used LS to gain insight into the kinetics of AtFtsZ2 assembly. We first varied protein concentration from 1 to 7 μM. The total FtsZ concentration in *Arabidopsis* chloroplasts has been roughly estimated at ∼3 μM (60). Assembly was initiated by adding 10 μM GTP, whereupon the LS signal began to increase (Fig. 2*A*). Similar to findings with bacterial FtsZs (55, 61), both the maximum extent and initial rate and of assembly trended higher with increasing AtFtsZ2 concentration (Fig. 2, *A* and *B*). At the higher concentrations assembly reached a plateau and then slowly decreased, consistent with GTP hydrolysis and depletion over time. The slow disassembly contrasts with the behavior of bacterial FtsZs, which disassemble rapidly upon GTP depletion (55, 56, 58).

To delve further into AtFtsZ2 assembly kinetics, we held the protein concentration at 5 μM and varied GTP concentration. As expected, the extent and initial rates of assembly generally correlated with GTP concentration (Fig. 2, *C* and *D*). Disassembly was not observed in reactions with a large excess of GTP (500 and 50 μM) during 2000 s of monitoring, but was at lower GTP concentrations. Extended monitoring over 5000 s in 5 μM GTP showed an eventual return of the LS signal to baseline, indicating complete disassembly (Fig. 2*C*, *inset*). TEM confirmed assembly and disassembly during the latter reaction (Fig. 2*E*), as well as differences in the extent of assembly over time in other reactions where nucleotide concentration was varied (Fig. S1).

It was surprising that 5 μM AtFtsZ2 assembled even in substoichiometric GTP (2.5 μM) (Fig. 2*C*, *pink trace*; Fig. S1, lower panels). We calculated when GTP should be used up in reactions initiated with 5 and 2.5 μM GTP based on the measured hydrolysis rate of 0.22 GTP min^−1^ FtsZ^−1^ (Fig. 1*C*; Table 1). Assembly continued beyond predicted GTP depletion (Fig. 2*C*, *arrowheads*) before decreasing very slowly, suggesting that AtFtsZ2 protofilaments are exceptionally stable. However, a few bacterial FtsZ studies have reported reduced specific GTPase activities at low GTP concentrations (61, 62, 63), and our GTPase measurements were performed in 500 μM GTP (Fig. 1*C*; Table 1). Therefore, we asked whether the apparent stability of AtFtsZ2 might instead be explained by reduced GTPase at low starting GTP concentrations or as GTP is depleted over time, but found no significant differences in specific activity over the range of starting concentrations used in our assembly experiments (2.5–500 μM; Fig. 2*F*). A caveat here is that our GTPase assay continuously converts GDP back to GTP to maintain GTP concentration (64), whereas in LS assembly assays GTP hydrolysis is accompanied by GDP accumulation, which could potentially slow hydrolysis and hence disassembly (56). But when we compared assembly of 5 μM AtFtsZ2 initiated with 2.5 μM GTP or with 2.5 μM GTP and 2.5 μM GDP, the kinetics of assembly and disassembly were very similar (Fig. S2). This suggests that reduced GTPase activity may not account for the stability of AtFtsZ2 protofilaments in the LS assays.

In contrast with AtFtsZ2, we could not detect assembly of AtFtsZ1 on its own by TEM, sedimentation, or LS (Figs. 1, *E*–*G* and 2C, *dark blue trace*). These results indicate that AtFtsZ1 either does not polymerize under our assembly conditions or forms polymers that are too small or too transient to be detected by these assays. However, AtFtsZ1 hydrolyzed GTP at a rate of 0.52 GTP min^−1^ FtsZ^−1^ (Fig. 1*C*; Table 1), though its activity did not exhibit a critical concentration (*i.e.*, the x intercept of the regression line relating GTP hydrolysis to AtFtsZ1 concentration was approximately 0) (Fig. 1*C*; Table 1). These results suggest that AtFsZ1 self-associates, perhaps in oligomers too short to be seen by TEM, with a high enough affinity to catalyze GTP hydrolysis. Collectively, our *in vitro* assays reveal that the two types of FtsZ in *Arabidopsis* have very different assembly properties when assayed separately.

### AtFtsZ1 constrains AtFtsZ2 assembly

**Figure 3:**
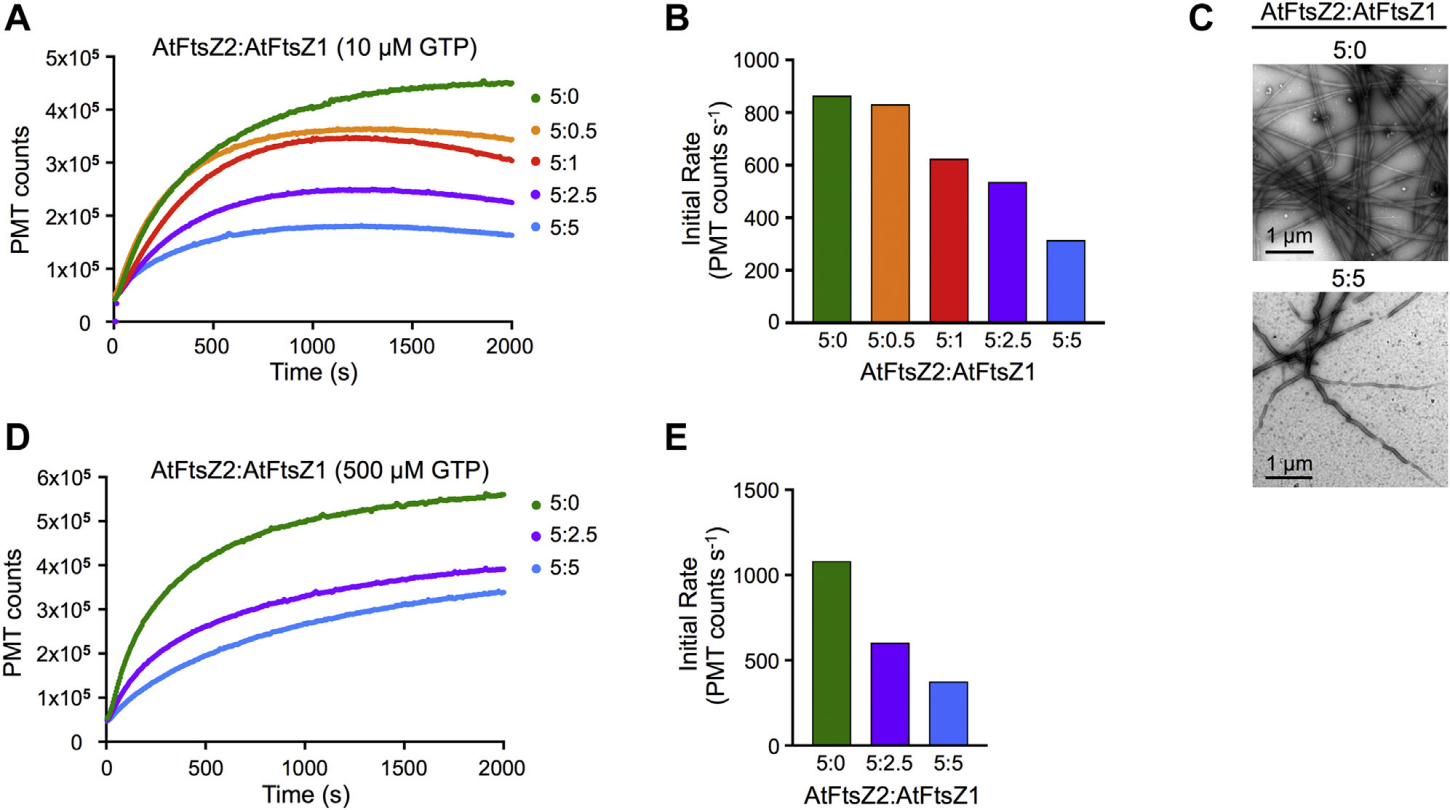
**AtFtsZ1 reduces assembly of AtFtsZ2.***A*, light scattering (LS) assays of 5 μM AtFtsZ2 mixed with AtFtsZ1 at various ratios, after addition of 10 μM GTP. *B*, initial rates of assembly for the LS traces in *A*. *C*, negative-stain TEM of 5:0 μM (*top*) and 5:5 μM (*bottom*) AtFtsZ2:AtFtsZ1 assembled for 2000 s after addition of 10 μM GTP. *D*, LS assays of 5 μM AtFtsZ2 mixed with AtFtsZ1 at the indicated ratios after addition of excess GTP (500 μM). *E*, initial rates of assembly for the LS traces in *D*.

Previous studies revealed that subunit exchange from AtFtsZ2 filaments assembled in heterologous yeast was very slow but that coassembly with AtFtsZ1 significantly increased subunit turnover, leading to the hypothesis that AtFtsZ1 reduces overall protofilament stability (24, 27). We employed LS to ask whether and how AtFtsZ1 would affect AtFtsZ2 assembly *in vitro*. AtFtsZ2 concentration was held constant at 5 μM while AtFtsZ1 was varied, and assembly was initiated with 10 μM GTP. As AtFtsZ1 concentration was increased, reductions in both the extent and initial rate of assembly were observed (Fig. 3, *A* and *B*). Some disassembly was also observed in all mixed reactions (Fig. 3*A*). TEM confirmed a reduction in assembly at equimolar AtFtsZ2:AtFtsZ1 (10 μM total protein) compared with 5 μM AtFtsZ2 alone (Fig. 3*C*). These results suggest that AtFtsZ1 reduces the overall assembly and abundance of protofilaments in a dose-dependent manner, which is consistent with a decrease in protofilament stability.

*In vitro* studies of bacterial FtsZs have shown that decreased protofilament stability is associated with higher GTPase activities (11, 12, 61, 65, 66, 67). To determine whether mixing AtFtsZ1 with AtFtsZ2 might stimulate GTP hydrolysis, we measured specific GTPase activities at the same AtFtsZ2:AtFtsZ1 ratios used in the LS assays, but found they were only very slightly higher than the specific activity of AtFtsZ2 alone and lower than that of AtFtsZ1 (Table 1; Fig. S3*A*). Therefore, the reduced extent and initial rates of assembly in the mixtures are not explained by stimulation of specific GTPase activity. However, they could reflect reduced nucleotide availability in the presence of more total AtFtsZ, and hence faster GTP depletion. To address this possibility, we carried out additional LS experiments in 500 μM GTP, where GTP should remain in large excess throughout the assay (Table S1). Decreases in protofilament abundance and initial rates of assembly were still observed as the ratio of AtFtsZ1 was increased (Fig. 3, *D* and *E*). These findings imply that these decreases are not due to reduced GTP availability but rather reflect a more direct effect of AtFtsZ1 on AtFtsZ2 assembly.

**Figure 4:**
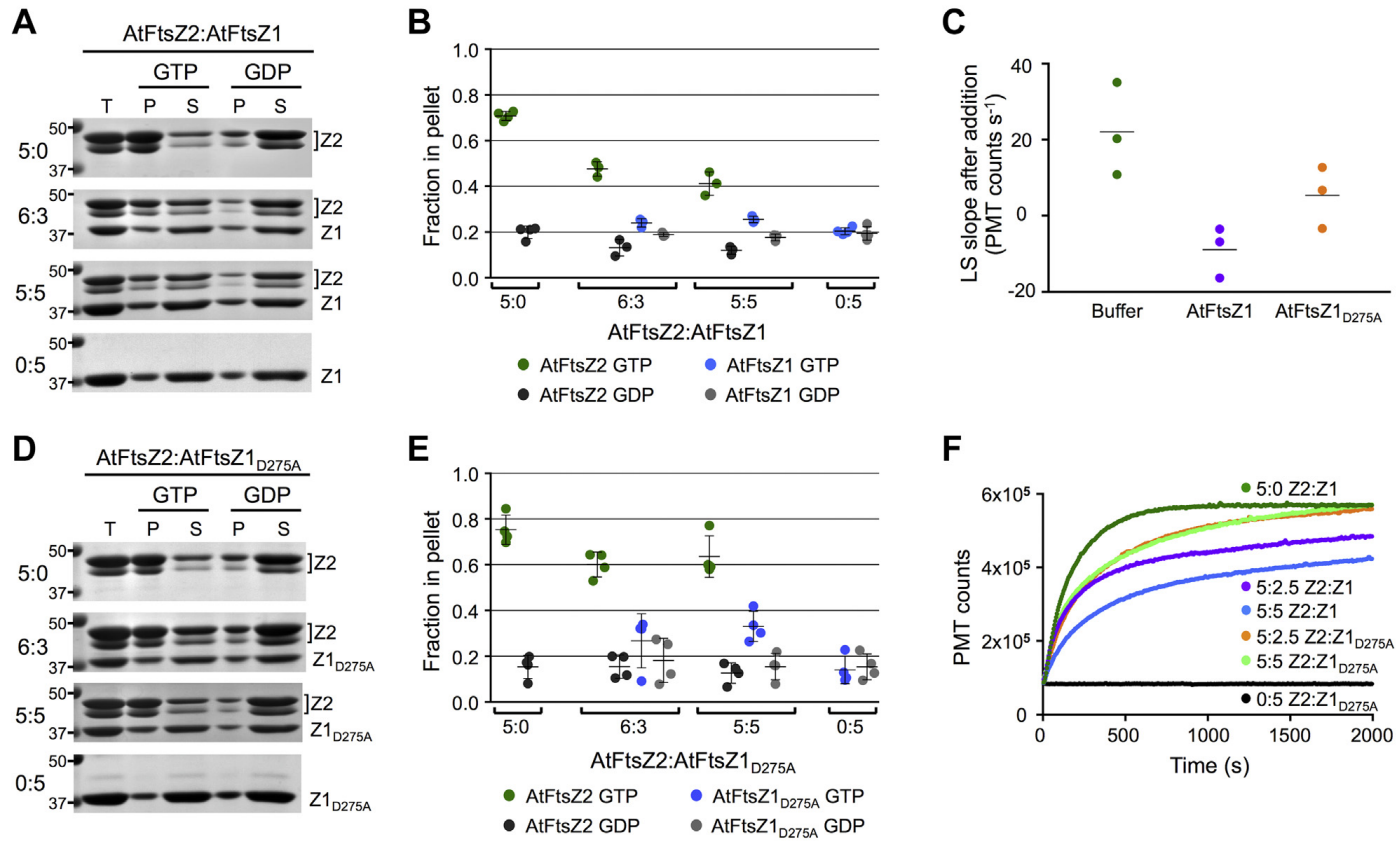
**AtFtsZ1 GTPase activity is required to reduce AtFtsZ2 assembly.***A*, *B*, *D* and *E*, sedimentation assays of AtFtsZ2 mixed with either AtFtsZ1 (*A* and *B*) or AtFtsZ1_D275A_ (*D* and *E*) at the indicated protein concentrations (μM). Assays were initiated by addition of 500 μM GTP or GDP and performed as described in Figure 1, *F* and *G*. *A* and *D*, SDS-PAGE of proteins in the total (T), pellet (P), and supernatant (S) fractions. Representative gels stained with Coomassie are shown. Markers (kDa) are indicated on the *left*. *B* and *E*, fraction of AtFtsZ in the pellet (n = 4 for all reactions except for AtFtsZ2:AtFtsZ1 mixed at 6:3 μM and 5:5 μM, where n = 3). The 5:0 μM and 0:5 μM data in *B* are repeated from Figure 1*G*. *C*, slopes of the light scattering (LS) traces of preassembled AtFtsZ2 after addition of buffer (*green*), AtFtsZ1 (*purple*), or AtFtsZ1_D275A_ (*orange*). Following each addition, the final concentration of AtFtsZ2, AtFtsZ1, AtFtsZ1_D275A_, and GTP were 5 μM, 2.5 μM, 2.5 μM, and 500 μM, respectively. See Fig. S4 for explanation of how slopes were determined. *F*, LS assays of 5 μM AtFtsZ2 (Z2) mixed with AtFtsZ1 (Z1) or AtFtsZ1_D275A_ (Z1_D275A_) at the indicated ratios. Assays were initiated by addition of 500 μM GTP.

To investigate the composition of the assemblies reported by LS in the mixed reactions, we used sedimentation assays to examine the relative amounts of AtFtsZ2 and AtFtsZ1 in the pellet fractions at different ratios in 500 μM GTP. The proportion of AtFtsZ2 in the pellet decreased as the ratio of AtFtsZ1 increased (Fig. 4, *A* and *B*, *green*), consistent with the reduction in LS (Figs. 3*D* and 4F). However, we did not detect a change in AtFtsZ1 in the pellet fraction in these reactions. This suggests that the same mechanism that prevents AtFtsZ1 from forming protofilaments on its own *in vitro* may also prevent stable integration of AtFtsZ1 into protofilaments in mixed reactions.

Toward addressing how AtFtsZ1 may be affecting AtFtsZ2, we tested the effect of adding AtFtsZ1 to preassembled AtFtsZ2. AtFtsZ2 was preassembled in excess GTP to a point where minimal additional assembly was occurring (∼1500 s; see Fig. 3*D* and Fig. S4). AtFtsZ1 was then added to a final AtFtsZ2:AtFtsZ1 ratio of 5:2.5 μM with 500 μM GTP, and LS was monitored for another 2000 s. The slope of the overall change in LS was compared with that obtained following addition of buffer as a control (see Experimental procedures). Fig. S4 shows a representative set of experiments. Addition of buffer or AtFtsZ1 caused an immediate but variable decrease in LS, presumably indicating some disruption of the preassembled AtFtsZ2. After addition of buffer, all the LS traces had positive slopes (Fig. 4*C*, *green*), likely representing gradual recovery of AtFtsZ2 assembly. In contrast, addition of AtFtsZ1 resulted in negative slopes (Fig. 4*C*, *purple*). The gradual decrease in LS after addition of AtFtsZ1 suggests that, as AtFtsZ2 subunits slowly dissociate from preassembled protofilaments, their net reassembly back onto protofilaments is diminished due to the presence of AtFtsZ1. These results are consistent with the decrease in assembly observed when AtFtsZ1 and AtFtsZ2 are mixed prior to initiating assembly (Figs. 3, *A*, *C* and *D* and 4, *A* and *B*, *green*), as likely occurs in chloroplasts based on *in vivo* expression data (60).

### AtFtsZ1 requires its GTPase activity to reduce AtFtsZ2 assembly

We have shown that AtFtsZ1 reduces AtFtsZ2 assembly even in excess GTP (Figs. 3*D* and 4, *A* and *B*). Previous FRAP data suggested that the ability of AtFtsZ1 to increase AtFtsZ2 subunit turnover depended on AtFtsZ1 GTPase activity (24). Therefore, we asked if AtFtsZ1 activity is required to reduce AtFtsZ2 assembly *in vitro*. To this end we purified AtFtsZ1_D275A_, the same mutant used in the FRAP study, which alters a highly conserved aspartate in the T7/synergy loop within the FtsZ active site (28). The equivalent mutation in *Escherichia coli* FtsZ (D212A) reduced GTPase to 7% of the wild-type activity (68).

The activity of AtFtsZ1_D275A_ was reduced to about 15% that of AtFtsZ1 (Table 1). AtFtsZ1_D275A_ behaved similarly to AtFtsZ1 in that assembly was not evident by sedimentation or LS assays (Fig. 4, *D*–*F*). However, increasing AtFtsZ1_D275A_ in mixture with AtFtsZ2 neither decreased the proportion of AtFtsZ2 in the pellet fraction (Fig. 4, *D* and *E*, *green*), nor did it decrease assembly to the same extent as AtFtsZ1 in LS assays (Fig. 4*F*). Preassembled AtFtsZ2 was also less affected by AtFtsZ1_D275A_ than by AtFtsZ1 (Fig. 4*C*; Fig. S4). These data show that AtFtsZ1 requires its GTPase activity to reduce the assembly of AtFtsZ2.

### The core regions of AtFtsZ1 and AtFtsZ2 confer key aspects of their unique biochemical behaviors

**Figure 5:**
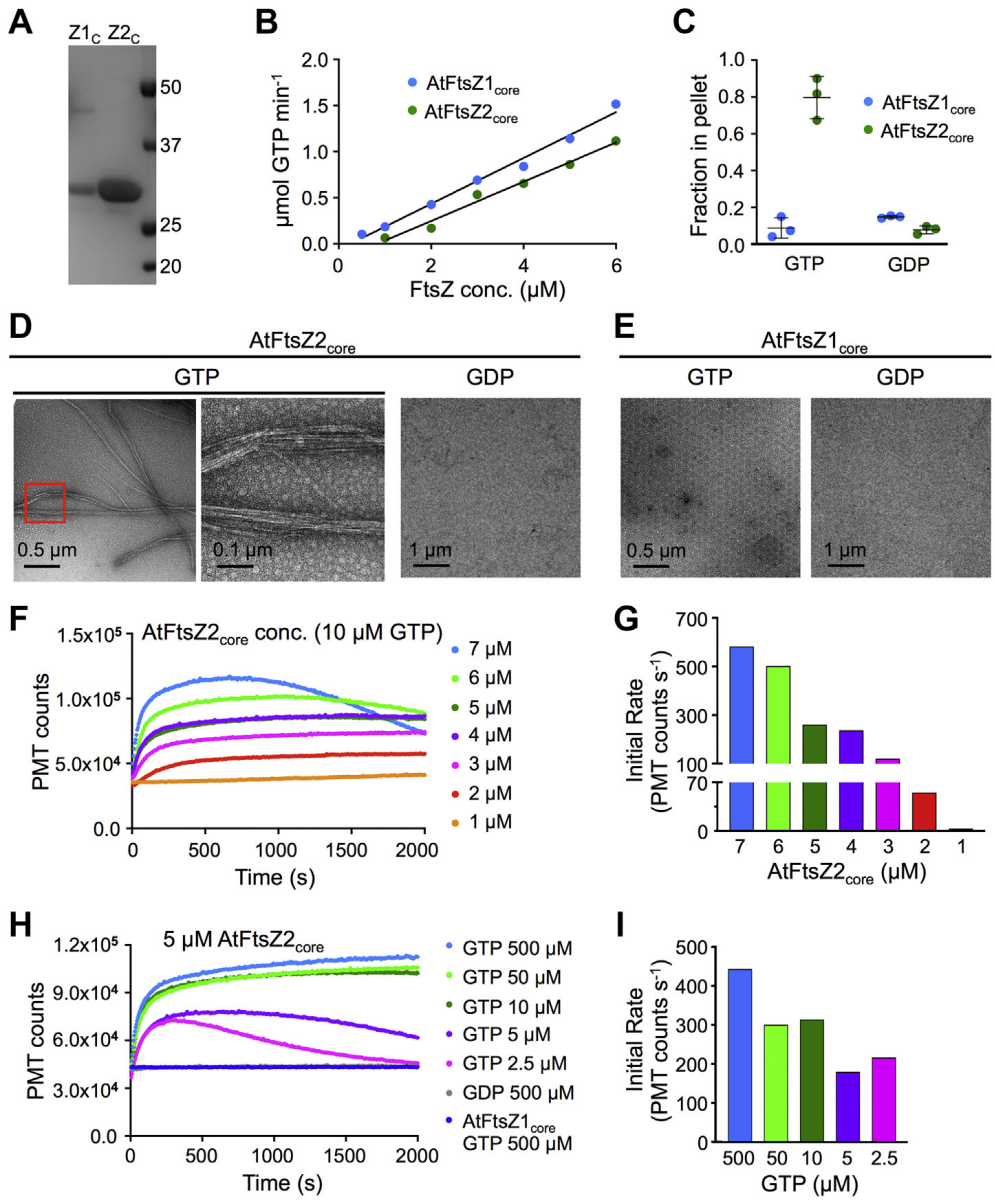
**The distinct assembly dynamics of AtFtsZ1 and AtFtsZ2 are determined primarily by their conserved core regions.***A*, SDS-PAGE of purified AtFtsZ1_core_ (Z1_c_) and AtFtsZ2_core_ (Z2_c_). The gel was stained with Coomassie. Markers (kDa) are shown on the *right*. *B*, GTPase activities assayed in 500 μM GTP at 25 °C at protein concentrations ranging from 0.5 to 6 μM for AtFtsZ1_core_ (*blue*) and from 1 to 6 μM for AtFtsZ2_core_ (*green*). A representative set of assays is shown. The GTPase activity is the slope of the regression line above the Cc. *C*, sedimentation assays of 3 μM AtFtsZ1_core_ (*blue*) or AtFtsZ2_core_ (*green*) after addition of 500 μM GTP or GDP. Assays were performed as described in Figure 1, *F* and *G*, centrifuged at 4 °C, and the fraction of protein in the pellet is shown (n = 3). A representative Coomassie-stained gel is shown in Figure 6*D*. *D* and *E*, negative-stain TEM of 3 μM AtFtsZ_core_ proteins incubated for 5 min after addition of 500 μM GTP or GDP. *D*, AtFtsZ2_core_. The region in the *red square* is shown at higher magnification in the *middle panel*. The average width of AtFtsZ2_core_ bundles assembled in GTP was 25.84 ± 13.1 nm (SD; n = 32; Fig. S5). *E*, AtFtsZ1_core_ in GTP or GDP. *F*, light scattering (LS) assays of AtFtsZ2_core_ at the indicated concentrations after addition of 10 μM GTP. *G*, initial rates of assembly for the LS traces in *F*. *H*, LS of 5 μM AtFtsZ2_core_ after addition of GTP or GDP at the indicated nucleotide concentrations. LS of 5 μM AtFtsZ1_core_ after addition of 500 μM GTP is also shown (*dark blue*). *I*, initial rates of assembly for the LS traces in *H*.

Structural analyses of FtsZs have indicated that their conserved core regions alone are likely responsible for GTP-dependent assembly and GTPase activity (5, 7, 13, 42, 43, 69, 70, 71, 72). However, to our knowledge no side-by-side comparison examining the assembly and GTPase activity of full-length and core variants has been reported for any FtsZ. We expressed and purified AtFtsZ1 and AtFtsZ2 composed of only the core regions (AtFtsZ1_core_ and AtFtsZ2_core_; Figs. 1*A* and 5A) to investigate their contributions to their different assembly properties. Confirming their functionality, we found that both core proteins possessed GTPase activity (Fig. 5*B*, Table 1). AtFtsZ1_core_ had about half the activity of AtFtsZ1 and also displayed a distinct Cc of 0.39 μM (Fig. 5*B*; Table 1), indicating the flanking regions may contribute to AtFtsZ1 GTPase activity. Neither the GTPase activity nor Cc of AtFtsZ2_core_ differed significantly from those of AtFtsZ2 (Table 1).

**Figure 6:**
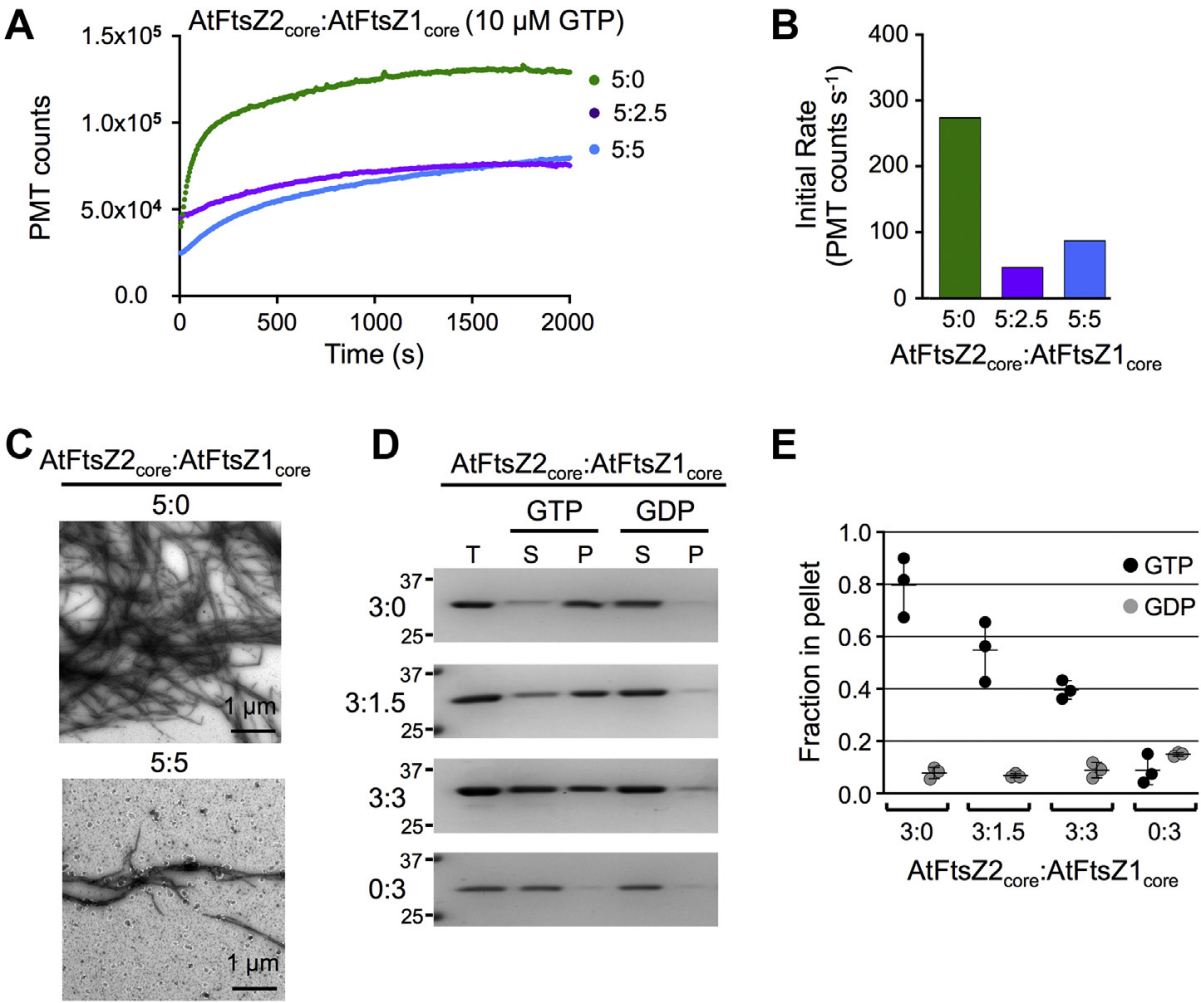
**AtFtsZ1**_**core**_**reduces the overall assembly of AtFtsZ**_**core**_**proteins.***A*, light scattering (LS) assays of 5 μM AtFtsZ2_core_ mixed with AtFtsZ1_core_ at the indicated ratios after addition of 10 μM GTP. *B*, initial rates of assembly for the LS traces in *A*. *C*, negative-stain TEM of 5:0 μM (*top*) and 5:5 μM (*bottom*) AtFtsZ2_core_:AtFtsZ1_core_ assembled for 2000 s after addition of 10 μM GTP. *D* and *E*, sedimentation assays containing AtFtsZ2_core_ mixed with AtFtsZ1_core_ at the indicated concentration ratios. Assays were initiated by addition of 500 μM GTP or GDP, performed as described in Figure 1, *F* and *G*, and centrifuged at 4 °C. *D*, SDS-PAGE of proteins in the total (T), supernatant (S), and pellet (P) fractions. Representative gels stained with Coomassie are shown. Markers (kDa) are indicated on the *left*. *E*, fraction of total AtFtsZ_core_ in the pellet (n = 3). The 3:0 and 0:3 μM data in *E* are repeated from Figure 5*C*.

We next examined the assembly properties of each core protein individually. AtFtsZ2_core_ exhibited GTP-dependent assembly in all assays (Figs. 5, *C*, *D*, *F* and *H* and 6D, *top gel image*). AtFtsZ2_core_ protofilaments formed bundles, but they appeared looser and were significantly thinner (25.84 ± 13.1 nm) than AtFtsZ2 bundles (60.15 ± 20.5 nm; *p* < 0.0001) (Figs. 1*D* and 5D; Fig. S5), suggesting the flanking regions contribute to lateral interactions between AtFtsZ2 protofilaments (73). In LS assays, the effects of protein and GTP concentration on assembly of AtFtsZ2_core_ were generally similar to their effects on AtFtsZ2 assembly (Figs. 2, *A*–*D* and 5, *F*–*I*). However, the maximum LS signals recorded for AtFtsZ2_core_ were ∼2.5- to 5-fold lower than for AtFtsZ2, consistent with the reduced bundling of AtFtsZ2_core_ protofilaments (Figs. 1*D* and 5D). The initial rates of AtFtsZ2_core_ assembly were also lower (Fig. 5*G*). AtFtsZ2_core_ protofilaments disassembled very slowly (Fig. 5, *F* and *H*), as confirmed by TEM (Fig. S6), indicating that the core region contributes substantially to AtFtsZ2 stability. Assembly of AtFtsZ1_core_ in 500 μM GTP could not be detected by sedimentation (Figs. 5*C* and 6D, *bottom gel image*), LS (Fig. 5*H*, *dark blue trace*), or TEM (Fig. 5*E*), suggesting that the assembly behavior of AtFtsZ1 is also largely a function of its core region.

We also examined the core proteins in mixture. Similar to the full-length proteins, mixing the two core AtFtsZs at different ratios did not increase specific GTPase activities (Table 1; Fig. S3*B*). LS showed a reduction in both the extent and initial rate of assembly with increasing AtFtsZ1_core_ (Fig. 6, *A* and *B*). Consistently, TEM revealed a lower abundance of protofilaments in the equimolar core mixtures (10 μM total protein) than with 5 μM AtFtsZ2_core_ alone (Fig. 6*C*). Although in sedimentation assays AtFtsZ1_core_ and AtFtsZ2_core_ could not be resolved by SDS-PAGE because they have nearly identical molecular masses (Fig. 5*A*), in excess GTP the proportion of total protein in the pellet fraction decreased as AtFtsZ1_core_ was increased (Fig. 6, *D* and *E*), strongly suggesting a reduction in assembly of AtFtsZ2_core_ based on the behavior of AtFtsZ2_core_ alone (Fig. 5). The overall similar behavior of the core and full-length proteins, including the ability of AtFtsZ1_core_ to reduce the assembly of AtFtsZ2_core_ without increasing overall GTPase activity, indicates that the assembly properties of both AtFtsZs are conferred largely by their conserved core regions.

## Discussion

Establishing the biochemical properties of FtsZ proteins *in vitro* is essential for understanding how these ancient, self-assembling proteins function within the diverse cell and organelle division machineries across the tree of life. Our results here represent the first comparative *in vitro* analysis of the mature, soluble forms of FtsZ1 and FtsZ2 from chloroplasts of green plants. The most important conclusions are that AtFtsZ2 assembles exceptionally stable protofilaments and bundles on its own and that AtFtsZ1 alters assembly kinetics by reducing the extent and rate of assembly in a manner that does not entail an increase in overall GTPase activity. Our findings suggest that restraining the self-assembly of FtsZ2 in chloroplasts as well as increasing Z-ring dynamics is a critical function of FtsZ1.

Unlike AtFtsZ2, which we confirmed forms protofilaments *in vitro* (52), AtFtsZ1 could not form detectable protofilaments alone under our assembly conditions (Figs. 1*E* and 5*E*). *In vitro* assembly of chloroplast FtsZ1 has been reported (51, 74, 75), but the functionality of the constructs used in the previous studies may have been compromised because they were truncated, had to be refolded from inclusion bodies, or contained the chloroplast transit peptide. An exception is FtsZ1 from *Medicago truncatula* (Mt). MtFtsZ1 lacking its transit peptide was soluble in *E. coli* and assembled into protofilament bundles and loops *in vitro* (76). The reason for the difference between AtFtsZ1 and MtFtsZ1 is unclear; it is possible that altering pH or ionic conditions, which we did not explore in the current study, could affect AtFtsZ1 assembly, as shown for bacterial FtsZs (11, 12, 61, 67, 77).

Generally, AtFtsZ2 behaves similarly to bacterial FtsZs except for its remarkable stability, revealed by its slow disassembly even after all the GTP should have been hydrolyzed (Fig. 2*C*, *arrowheads*), and by its assembly even in a substoichiometric concentration of GTP (Fig. 2*C*), the kinetics of which were unaltered by inclusion of equimolar GDP (Fig. S2). While bundling may contribute to AtFtsZ2’s stability, various bacterial FtsZs also undergo considerable bundling (9, 55, 77, 78). Our current data rather suggest that the stability of AtFtsZ2 is an intrinsic biochemical property of its homopolymers.

Notably, the extreme stability of AtFtsZ2 polymers and lack of detectable AtFtsZ1 assembly *in vitro* are reminiscent of their mutant phenotypes in Arabidopsis. In *atftsZ1* mutants, AtFtsZ2 is localized in excessively long filaments and spirals, suggesting these structures are hyperstabilized. Chloroplasts in these mutants are larger, fewer in number, and much more variable in size than in wild type but still divide to some degree, indicating that AtFtsZ2 retains partial functionality *in vivo* without AtFtsZ1 (6, 39, 40). In contrast, in *atftsZ2* mutants AtFtsZ1 is detected in very short filaments and punctate structures, suggesting that it forms less stable assemblies in the absence of AtFtsZ2, and chloroplast division is much more drastically impaired (6, 40, 79). Because of their abnormal chloroplast morphologies, both mutants are also more susceptible to photodamage than wild-type plants (80, 81), stressing the importance of both FtsZs for physiological function. Based on the mutant phenotypes and our current *in vitro* findings, we postulate that FtsZ2 is the main orchestrator of chloroplast division (which is also consistent with its unique function in Z-ring tethering to the membrane [40, 49, 50]) and that the primary function of FtsZ1 is to counteract the extreme stability of AtFtsZ2 to enhance chloroplast Z-ring dynamics.

While investigating the individual features of AtFtsZ1 and AtFtsZ2 is necessary for understanding how each contributes to chloroplast division, the fact that these proteins invariably colocalize *in vivo*, both to Z rings and to the aberrant, morphologically diverse FtsZ structures seen in numerous chloroplast division mutants (6, 15, 50, 79), strongly suggests that coassembly is their physiologically relevant state. Our work here examining their *in vitro* biochemical properties in mixture is therefore important for understanding how they cooperate *in vivo*. TerBush and Osteryoung (24) found that coassembly of AtFtsZ1 with AtFtsZ2 increased the otherwise very slow turnover of AtFtsZ2 subunits from protofilaments. The stability of AtFtsZ2 *in vitro* (Fig. 2) and decrease in its assembly as AtFtsZ1 is increased in LS assays (Figs. 3 and 4*B*) agree with these results and imply their direct interaction in mixture, as does the gradual decrease in LS when AtFtsZ1 was added to preassembled AtFtsZ2 (Fig. 4*C*; Fig. S4). However, our sedimentation experiments, while not ruling out coassembly, did not provide direct evidence of coassembly because we could not detect an increase in AtFtsZ1 in the pellet fraction as AtFtsZ1 was increased (Fig. 4*B*). At present we do not know the explanation. Regardless, our *in vitro* analysis suggests that the AtFtsZ1-induced enhancement of AtFtsZ2 dynamics observed in yeast (24, 27) is not due to a significant stimulation of GTPase activity, although AtFtsZ1 GTPase activity is still required (24) (Fig. 4). We propose that AtFtsZ1 counterbalances the stabilizing function of AtFtsZ2 through a different mechanism that both promotes AtFtsZ2 subunit turnover from protofilaments and limits AtFtsZ2 self-assembly. One possibility hypothesized by TerBush and Osteryoung (24) is that coassembly of AtFtsZ1 with AtFtsZ2 decreases the affinity between subunits, promoting protofilament fragmentation and increasing the number of subunits available for exchange from protofilament ends. Our results here are consistent with this model.

Our present *in vitro* study of FtsZ1 and FtsZ2 from the chloroplasts of green plants complements our similar study of chloroplast FtsZA and FtsZB from the red alga *Galdieria sulphuraria* (Gs) (54). FtsZA is similar to FtsZ2 in that it retains the CTP, while FtsZB, like FtsZ1, lacks it (45, 47) (Fig. 1*A*). In LS assays, 10 μM GsFtsA assembled in 50 μM GTP and reached a plateau over 40 to 100 min before slowly disassembling even though its steady-state GTPase would predict complete GTP hydrolysis after 7 min. Thus GsFtsZA exhibited an extended lifetime similar to that of AtFtsZ2. At present we do not know the mechanism; perhaps fragmentation at GDP-bound interfaces and subunit dissociation from protofilament ends occur more slowly than in bacterial FtsZ protofilaments (23). When GsFtsZA and GsFtsZB were mixed, assembly was initially greater than with GsFtsZA alone, but disassembly occurred much more rapidly (54). In a separate study, FRAP experiments showed that GsFtsZA filaments were much less dynamic than GsFtsZB filaments when expressed separately in yeast, but that GsFtsZA subunit exchange was strongly enhanced by coexpression with GsFtsZB (25). Interestingly, the GsFtsZ constructs used in both studies consisted of only their conserved core regions, suggesting that these regions contribute substantially to their distinct dynamic behaviors, as shown here for AtFtsZ1 and AtFtsZ2.

The assembly dynamics of bacterial FtsZs are thought to be dominated by treadmilling, which guides the peptidoglycan (cell wall) synthesis machinery around the cell division site. Treadmilling requires protofilament polarity, where subunits preferentially assemble onto one end of the protofilament and dissociate from the other due to a difference in the net rates of subunit addition and loss on the two ends (19, 21, 22, 23, 82). Because of this polarity, if GTP is depleted, GTP-dependent assembly at one end of the protofilament is halted while disassembly continues at the other end, resulting in rapid protofilament disassembly. The chloroplast FtsZs behave very differently, and we have so far not been able to fit their dynamics into a recent kinetic model of treadmilling (23). However, while many chloroplasts lack a peptidoglycan wall, they do have a highly complex division machinery whose operation depends on Z-ring function (2, 3, 4). Experiments on chloroplast Z rings reconstituted in yeast suggested that AtFtsZ2 protofilaments may have some polarity despite their stability, but that polarity was greatly increased by coassembly with AtFtsZ1 (27). It is conceivable that the decrease in protofilament stability and enhanced turnover dynamics induced by FtsZ1 in plants and FtsZB in red algae (Fig. 3) (24, 25, 54) might also promote treadmilling, which could facilitate Z-ring constriction and chloroplast division by this complex apparatus.

It is intriguing that chloroplasts in photosynthetic organisms separated by over a billion years of evolution (83) both possess one FtsZ with exceptional stability and a second that counterbalances this stability. This underscores the importance of these complementary functions in the overall regulation of chloroplast FtsZ dynamics.

## Experimental procedures

### Production and purification of recombinant FtsZ proteins

Full-length *A. thaliana* AtFtsZ1-1 (AtFtsZ1; At5g55280) and AtFtsZ2-1 (AtFtsZ2; At2g36250) without their predicted transit peptides (Fig. 1*A*) were expressed with C-terminal 6x His (His_6_) tags in pDB328 (41). Construction of these expression vectors was described previously (51). To construct the vectors for expression of AtFtsZ1_core_ and AtFtsZ2_core_ (Fig. 1*A*), fragments encoding the conserved core regions of AtFtsZ1 (aa 71–376) and AtFtsZ2 (aa 118–423) bearing C-terminal His_6_ tags were PCR-amplified from the plasmids used for expression of full-length AtFtsZ1 and AtFtsZ2 (51) using the primer pairs AT72F (TTTTTTCCATGGAATCTGCGAGAATTAAGGTGATTGGTGTCGGT) and AT40R (TTTTTTCTCGAGCTAATGATGATGATGATGATGGCCTGTGGCGATTATCGTTACATGAAT), and AT73F (TTTTTTCCATGGAGGCGAGGATTAAGGTTATTGGTGTG) and AT42R (TTTTTTCTCGAGTTAATGATGATGATGATGATGACCCGTAGCTATCAGGGTTATGCTTAC), respectively. The amplified fragments were cloned into the NcoI and XhoI sites of the expression vector pLW01 (84), creating the vectors pAT2620 encoding AtFtsZ1_core_ and pAT2621 encoding AtFtsZ2_core_. AtFtsZ1_D275A_ was amplified using two sets of primers LY251F (GAAGGAGATATACATATGAGGTCTAAGTCGATGCGATTGAGG) with KP82R (CTTTCATGACTGCCTTCACTGCTGCAAAATCTACATTGACT) and KP81F (TGTAGATTTTGCAGCAGTGAAGGCAGTCATGAAAG) with LY254R (GTTAGCAGCCGGATCCTCAGTGGTGGTGGTGGTGGTGGAAGAAAAGTCTACGGGGAGAAG) resulting in two fragments now possessing the desired point mutation. Gibson assembly (85) was then used to insert the two fragments into pET11b (86) digested with NdeI and BamHI resulting in the vector pKO2505. The expression vectors were transformed into DE3 Rosetta cells (Novagen) overexpressing the *E. coli ftsQAZ* operon (87).

Olson *et al.* (51) found that the mature forms of AtFtsZ1 and AtFtsZ2 lacking their predicted chloroplast transit peptides could be expressed following Isopropyl-β-D-1-thiogalactopyranoside (IPTG) induction and growth for 3 h at 37 °C, but most of the expressed protein was in inclusion bodies, and experiments had to be performed on refolded proteins. We revisited expression conditions for the same His-tagged AtFtsZ1 and AtFtsZ2 constructs and *E. coli* strain described by Olson *et al.* (51) to identify conditions that would yield soluble protein. Bacterial strains expressing the AtFtsZ proteins were grown overnight at 37 °C and subcultured into fresh LB. The cultures were allowed to grow until A_600_ reached ∼0.6 to 0.8 and were then cold-shocked for 10 min in an ice bath. IPTG was then added to a final concentration of 0.6 mM, and the culture was grown for 36 to 42 h at 14 °C. Cells were collected by centrifugation and resuspended in 20 ml low salt buffer (LSB; 50 mM Tris pH 7.5, 50 mM NaCl, 10% glycerol), and the pellet was frozen at −80 °C.

Purification of all FtsZ proteins was conducted utilizing the His_6_ tag. Frozen cell suspensions were thawed and cells were initially lysed chemically with 1 mg/ml Lysozyme (Lab scientific) for 30 min at 4 °C with rotation and then further ruptured by sonication. Lysate containing the soluble protein was collected after centrifugation at 10,000*g* for 25 min at 4 °C and loaded onto a Ni-NTA column (Qiagen) at room temperature. The column was then washed with increasing concentrations of imidazole (10 mM–50 mM) in LSB, and ultimately the protein was eluted in 300 mM imidazole in LSB. Fractions containing purified protein were then pooled and dialyzed into LSB at 4 °C, aliquoted, and stored at −80 °C. Protein concentrations were determined prior to each use following centrifugation at 80,000*g* using the bicinchoninic acid assay (Thermo Scientific) and implementing a 20% correction factor (51, 62). The concentrations of purified proteins ranged from 25 to 85 μM with the exception of AtFtZ1 and AtFtsZ1_D275A_, which ranged from 10 to 20 μM. Efforts to further concentrate either AtFtsZ1 or AtFtsZ1_D275A_ resulted in precipitation of protein.

### GTPase measurement

A regenerative GTPase assay modified slightly from Ingerman and Nunnari (64) was used to determine GTPase activities of the FtsZs. The assay utilizes a reaction that allows for the continuous regeneration of GDP to GTP through the consumption of a single NADH per regeneration of GTP. The assay itself measures the depletion of NADH at 340 nm. Prior to GTPase measurements, the proteins were centrifuged at 80,000*g* for 30 min at 4 °C and subsequently quantified as described above. To initiate each 200 μl reaction, the desired concentration of protein in LSB was dispensed into a well of a 96-well plate and the volume was adjusted to 137.4 μl for all reactions with additional LSB. Next, 42.6 μl of GTPase reaction buffer with magnesium (4.7 mM phosphoenolpyruvate [Sigma #P7002], 1.9 mM NADH [Sigma #N8129], 500 mM HEPES-KOH, pH 7.5, 50 mM MgSO_4_, 1 M KCL, with 5 μl pyruvate kinase/lactose dehydrogenase [Sigma #P0294]) were added, resulting in an overall reaction containing 1 mM phosphoenolpyruvate, 0.4 mM NADH, and 20 U/ml pyruvate kinase/lactate dehydrogenase in 50 mM HEPES-KOH, pH 7.5, 5 mM MgSO_4_, 100 mM KCL. Lastly, the reaction was initiated by the addition of 20 μl GTP (Sigma #G8877) in LSB to a final concentration of 500, 100, 10, 5, or 2.5 μM GTP, and absorbance at 340 nm was measured for 300 min (Molecular Devices, SpectraMax [M2]). The velocity of hydrolysis was determined utilizing a manipulation of Beer’s law as follows:

$$\left(moles\;of\;GTP\;hydrolyzed|min)=\;(\Delta A_{340}|{\;}_{\varepsilon NADH}L\;\cdot V_{a}\right),$$

where $\Delta A_{340}$ is the slope of NADH depletion; εNADH is the extinction coefficient of NADH at 340 nm (6220 M^−1^ cm^−1^); *L* is the path length of the well (0.4 cm); and *V*_*a*_ is the reaction volume (200 μl). The determined velocity of GTP hydrolysis (μmol GTP min^−1^) was then plotted as a function of the total FtsZ concentration in each reaction, and the slope of the linear range was taken as the GTPase activity (GTP min^−1^ FtsZ^−1^) for each set of reactions. The X-intercept was taken as the critical concentration (Cc) of FtsZ required for GTPase activity. Rates are shown as an average of multiple replications of assays performed on multiple protein preparations on various days (n = 3–10; Table 1), demonstrating the consistency of the results. Errors are shown as SD. For GTPase activity of AtFtsZ2 at different GTP concentrations, the velocity of GTP hydrolysis (μmol GTP min^−1^) at each GTP concentration shown in Figure 2*F* was determined at 1, 2, 3, 4, 5, 6, and 7 μM AtFtsZ2. The velocity at each GTP concentration was then plotted as a function of AtFtsZ2 concentration and the slope of the linear range was taken as the GTPase activity (GTP min^−1^ FtsZ^−1^).

### Assembly buffer

Assembly experiments monitored by LS, sedimentation, and TEM were conducted in HMK buffer: 50 mM HEPES-KOH, pH 7.5, 5 mM MgSO_4_, 100 mM KCl. All reactions were initialed by addition of nucleotide.

### Electron microscopy

Assembly reactions for TEM were carried out in 20 to 100 μl total volumes. Prior to assembly experiments, the proteins were centrifuged at 80,000*g* for 30 min at 4 °C and subsequently quantified as described above. The desired concentration of protein in LSB was first added to each reaction tube and allowed to warm to room temperature prior to initiation of each reaction. A 1:10 dilution of the 10X HMK buffer (500 mM HEPES-KOH, pH 7.5, 50 mM MgSO_4_, 1 M KCl) was first added and a 10X nucleotide solution in LSB was then added to obtain the desired final 1X nucleotide concentration. The reaction was allowed to assemble at room temperature for the desired time and 5 μl of the reaction was pipetted onto to a carbon-coated 400-mesh copper grid prepared in our laboratory. The buffer was then wicked away and the sample was washed with 5 μl water, which was also wicked away quickly. A 2% uranyl formate stain was then applied and quickly wicked away. Protofilament structures were visualized with a JEOL100 CXII (Japan Electron Optics Laboratories) TEM or a JEOL 1400 Flash transmission electron microscope at magnifications from 6000 to 100,000×. Filament widths were measured using ImageJ (88).

### 90° light scattering

90° LS assays were performed at room temperature as described previously (51, 54) with a fluorescence spectrophotometer (Photon Technology International) equipped with a model 814 photomultiplier utilizing the digital mode set at 1000 V. The following parameters were used to conduct the assembly assays; the lamp was set to 5 mm with 1 mm excitation and 1.5 mm emission slit widths. The excitation and emission wavelengths were both 350 nm. Prior to the experiments, the proteins were centrifuged at 80,000*g* for 30 min at 4 °C and subsequently quantified as described above. LS assays were performed in 150 μl total volume. The desired concentration of protein in 120 μl LSB was first added to each reaction tube and allowed to warm to room temperature for approximately 20 min. Next, 15 μl of 10X HMK buffer was added, followed by 15 μl of 10X nucleotide in LSB. The reaction was transferred to a 0.5 cm quartz cuvette, and the LS signal (PMT counts) was recorded every 5 s for the entirety of the assay. All assembly reactions were performed at room temperature and repeated at least twice with different protein preparations and similar results were obtained. Only data obtained on the same day during the same experiment are plotted together. It should be noted that some minor variability was observed, most likely due to slight variations in room temperature and between protein preparations.

The initial rate of assembly was determined by manually trimming the LS plot and using Prism Graphpad to determine the slope of the initial portion of the plot reported in PMT s^−1^. Stringency of the selected initial rate was achieved by trimming the LS plot until an R^2^ value of >0.95 was obtained. An initial rate of assembly was determined for each LS trace (minimum of two replicates) and the trends were similar. The initial rates shown in each figure correspond to the LS assays presented in that figure.

### Effect of AtFtsZ1 and AtFtsZ1_D275A_ on preassembled AtFtsZ2

AtFtsZ2 was first assembled alone and monitored by 90° LS as described above. This preassembly was performed in volumes between 88 and 99.7 μl, where between 58 and 69.7 μl AtFtsZ2 in LSB was first added to the reaction tube and allowed to warm to room temperature for approximately 20 min. Next, the reaction was transferred to the 0.5 cm quartz cuvette and 15 μl of 10X HMK buffer was added, followed by 15 μl of 10X GTP in LSB. The resulting concentration of AtFtsZ2 was between 7.5 and 8.6 μM, and the concentration of GTP was between 752 and 855 μM. The LS signal (PMT counts) was monitored until it reached a state where minimal additional assembly was occurring (∼1500 s). Between 50.3 and 62 μl AtFtsZ1 or AtFtsZ1_D275A_ in LSB or LSB alone (buffer) was then added, resulting in final AtFtsZ2 and GTP concentrations of 5 μM and 500 μM, respectively, in all reactions, and in the mixtures a final concentration of AtFtsZ1 or AtFtsZ1_D275A_ of 2.5 μM. LS was monitored for an additional 2000 s. The same volume of buffer, AtFtsZ1, or AtFtsZ1_D275A_ was used for each treatment within an experiment. Experiments were performed at room temperature and repeated three times with similar results. Prism Graphpad was used to determine the slope of each LS trace after addition of buffer, AtFtsZ1 or AtFtsZ1_D275A_ (see Fig. S4). All slopes are plotted in Figure 4*C*.

### Sedimentation assays

Prior to sedimentation assays the proteins were centrifuged at 80,000*g* for 30 min at 4 °C and subsequently quantified as described above. The desired concentration of protein in LSB was first added to each reaction tube and allowed to warm to room temperature prior to initiation of each reaction. All reaction volumes were the same to allow for direct comparisons between samples. One-tenth volume of the 10X HMK buffer was then added followed by addition of nucleotide at a 10X concentration, allowing for the desired final concentration. FtsZs and nucleotides were incubated at room temperature for 30 min and centrifuged at 80,000*g* in an S100AT4 607 rotor (ThermoFisher Scientific) for 30 min at either 25 °C or 4 °C as indicated in the figure legends. The supernatant was collected and the pellet was then resuspended in the same volume of LSB. A sample of the protein not centrifuged was used as the total protein control. The total protein, supernatant, and pellet samples were analyzed by SDS-PAGE and quantified by densitometry using ImageJ software (88). Sedimentation assays were carried out three or four times as indicated in figure legends, and the proportions of protein in the pellet fractions (density of pellet/density of pellet + density of supernatant) are reported. At least two different protein preparations were used for all experiments and similar results were obtained.

### Statistical analysis

All statistical analyses were performed using Prism Graphpad software. All plots were also generated with Prism Graphpad, where reported errors represent standard deviation (SD). All *p* values represent unpaired *t*-tests, unless stated otherwise.

## Data availability

Most data are contained within the figures and tables except where it’s explicitly stated that representative data are shown. Raw data are available upon request from the corresponding author.

## Supporting information

This article contains supporting information.

## Conflict of interest

The authors declare that they have no conflicts of interest with the contents of this article.
