# Supplementary material for: The Arabidopsis thaliana chloroplast division protein FtsZ1 counterbalances FtsZ2 filament stability in vitro
Source: J Biol Chem. 2021 Apr 2;296:100627. doi: 10.1016/j.jbc.2021.100627 (PMC8142252; doi:10.1016/j.jbc.2021.100627)
Supplement: Supplemental Figures S1–S6 and Table S1 [file mmc1.pdf]

The *Arabidopsis thaliana* chloroplast division protein FtsZ1 counterbalances FtsZ2 filament stability *in vitro*

Katie J. Porter, Lingyan Cao, Yaodong Chen, Allan D. TerBush, Cheng Chen, Harold P. Erickson, Katherine W. Osteryoung

Supporting Information

Fig. S1. Negative-stain transmission electron microscopy of AtFtsZ2.

Fig. S2. Light scattering of AtFtsZ2 assembled with GTP alone or with GTP and GDP.

Fig. S3. GTPase assays of AtFtsZ and AtFtsZ$_{core}$ proteins mixed at different ratios.

Fig. S4. Effect of AtFtsZ1 and AtFtsZ1$_{D275A}$ on preassembled AtFtsZ2 monitored by light scattering.

Fig. S5. Widths of AtFtsZ2 and AtFtsZ2$_{core}$ protofilament bundles.

Fig. S6. Negative-stain transmission electron microscopy of AtFtsZ1$_{core}$ and AtFtsZ2$_{core}$.

Table S1. Predicted GTP concentrations remaining after 2000 seconds in light scattering assays shown in Figure 3D and associated parameters.

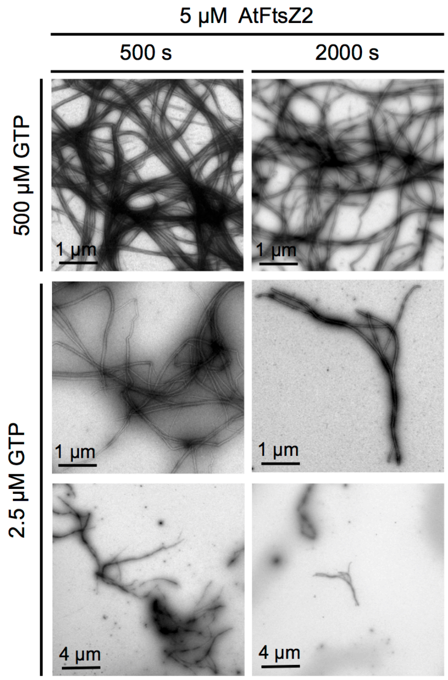

Fig. S1. Negative-stain transmission electron microscopy of AtFtsZ2. 5 µM AtFtsZ2 was incubated for 500 s (left) or 2000 s (right) after addition of 500 µM GTP (top) or 2.5 µM GTP (middle and bottom). Images for the latter reaction are shown at two magnifications.

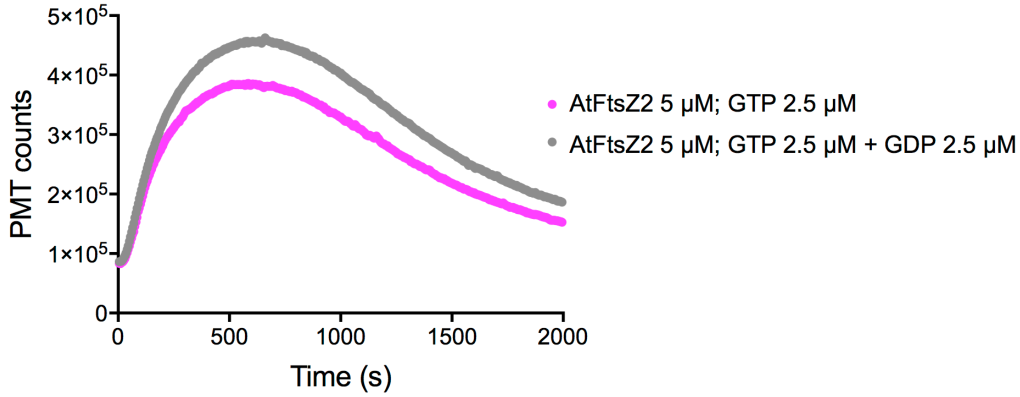

Fig. S2. Light scattering of AtFtsZ2 assembled with GTP alone or with GTP and GDP. Assembly of 5 μM AtFtsZ2 was initiated with either 2.5 μM GTP (pink) or 2.5 μM GTP and 2.5 μM GDP (gray) and monitored by light scattering. The assembly assay was performed at room temperature and repeated twice with similar results.

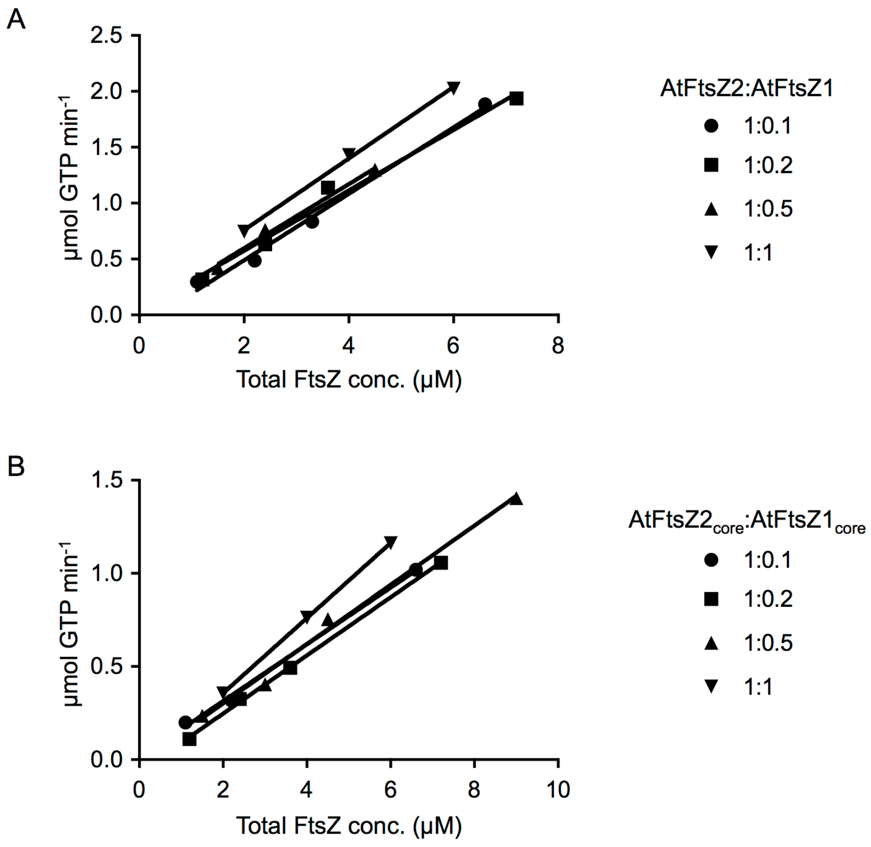

Fig. S3. GTPase assays of AtFtsZ and AtFtsZ$_{core}$ proteins mixed at different ratios. Activities were assayed at the indicated ratios at 25° C in 500 µM GTP. The GTPase activity is the slope of the regression line above the Cc. Representative GTPase activities are shown for *A*, AtFtsZ2 mixed with AtFtsZ1, and *B*, AtFtsZ2$_{core}$ mixed with AtFtsZ1$_{core}$.

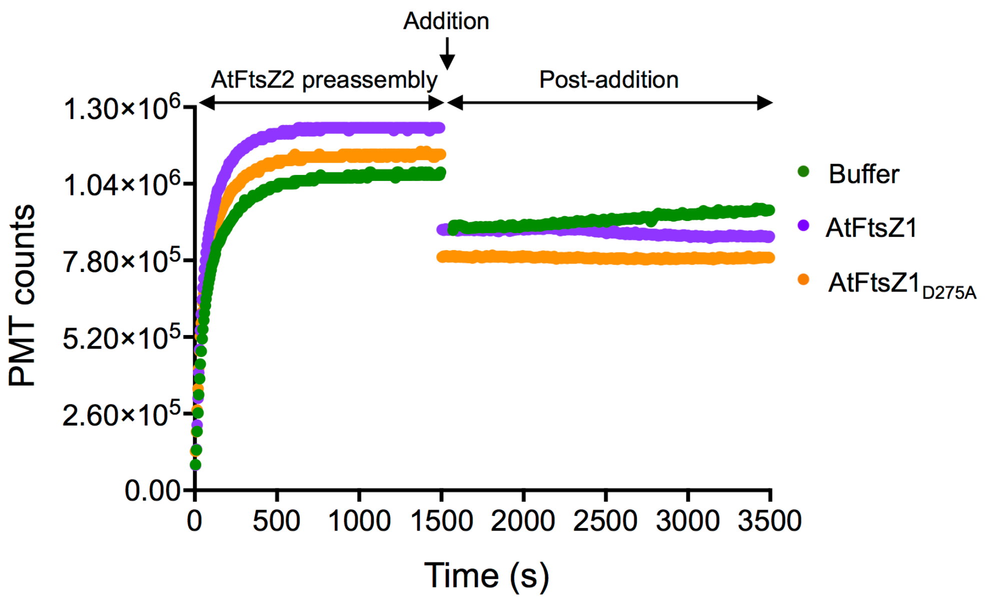

Fig. S4. Effect of AtFtsZ1 and AtFtsZ1$_{D275A}$ on preassembled AtFtsZ2 monitored by light scattering. AtFtsZ2 (7.5-8.6 µM) was assembled in a large excess of GTP at room temperature for approximately 1500 s (AtFtsZ2 preassembly; see methods). Buffer, AtFtsZ1 or AtFtsZ1$_{D275A}$ was then added (Addition), resulting in final concentrations of AtFtsZ2, AtFtsZ1, AtFtsZ1$_{D275A}$ and GTP of 5 µM, 2.5 µM, 2.5 µM and 500 µM, respectively. Light scattering was monitored for another 2000 s (Post-addition). The slope of each trace over the entire post-addition period was determined and all slopes are reported in Fig. 4*C*. A representative experiment is shown here, in which all data were obtained on the same day.

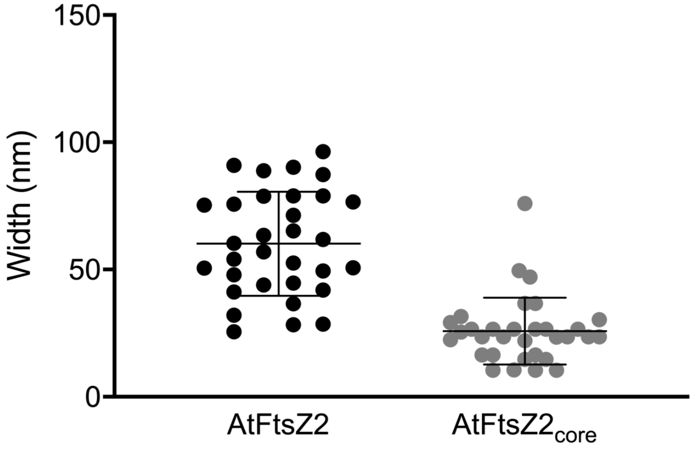

Fig. S5. Widths of AtFtsZ2 and AtFtsZ2$_{core}$ protofilament bundles. Scatter plots of widths of AtFtsZ2 and AtFtsZ2$_{core}$ bundles show means ± SD (n=32). Widths of AtFtsZ2 and AtFtsZ2$_{core}$ bundles were statistically different ($p<0.0001$; unpaired t-test).

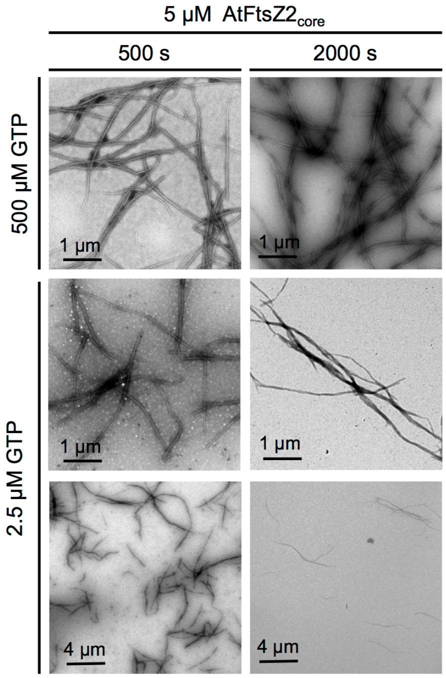

Fig. S6. Negative-stain transmission electron microscopy of AtFtsZ1$_{core}$ and AtFtsZ2$_{core}$. 5 µM AtFtsZ2$_{core}$ was incubated for 500 s (left) or 2000 s (right) after addition of 500 µM GTP (top) or 2.5 µM GTP (middle and bottom). Images for the latter reaction are shown at two magnifications.

Table S1. Predicted GTP concentrations remaining after 2000 seconds in light scattering assays shown in Figure 3D, and associated parameters. GTPases activities reported as GTP min^{-1} $FtsZ^{-1}$ are from Table 1, where they were measured using a regenerative system that maintained the GTP concentration at 500 µM, and were converted to GTP s^{-1} $FtsZ^{-1}$. AtFtsZ concentration (conc.) above the critical concentration (Cc) was calculated by subtracting the Cc reported in Table 1 from the total FtsZ concentration in the reaction[1]. GTP concentration remaining after 2000 s was calculated by first multiplying the GTPase activity in GTP s^{-1} $FtsZ^{-1}$ by the AtFtsZ conc. above Cc by 2000 s, and subtracting that value from the starting GTP concentration in the reaction (500 µM). Fold excess GTP was calculated by dividing GTP remaining after 2000 s by AtFtsZ conc. above the Cc.

AtFtsZ2:AtFtsZ1 (µM:µM)	AtFtsZ conc. above the Cc (µM)	GTPase activity (GTP $min^{-1}$$FtsZ^{-1}$)	GTPase activity (GTP $s^{-1}$$FtsZ^{-1}$)	GTP remaining after 2000 s (µM)	Fold excess GTP
5:0	4.64	0.22	0.0037	466	100
5:2.5	7.46	0.27	0.0045	433	58
5:5[1]	10	0.36	0.006	380	38

[1]A Cc of 0 µM was used for AtFtsZ2:AtFtsZ1 (5:5).
